# Common and Unique Contributions of Decorin-Binding Proteins A and B to the Overall Virulence of *Borrelia burgdorferi*

**DOI:** 10.1371/journal.pone.0003340

**Published:** 2008-10-03

**Authors:** Yanlin Shi, Qilong Xu, Sunita V. Seemanaplli, Kristy McShan, Fang Ting Liang

**Affiliations:** Department of Pathobiological Sciences, Louisiana State University, Baton Rouge, Louisiana, United States of America; University of California Merced, United States of America

## Abstract

As an extracellular bacterium, the Lyme disease spirochete *Borrelia burgdorferi* resides primarily in the extracellular matrix and connective tissues and between host cells during mammalian infection, where decorin and glycosaminoglycans are abundantly found, so its interactions with these host ligands potentially affect various aspects of infection. Decorin-binding proteins (Dbps) A and B, encoded by a 2-gene operon, are outer surface lipoproteins with similar molecular weights and share approximately 40% identity, and both bind decorin and glycosaminoglycans. To investigate how DbpA and DbpB contribute differently to the overall virulence of *B. burgdorferi*, a *dbpAB* mutant was modified to overproduce the adhesins. Overproduction of either DbpA or DbpB resulted in restoration of the infectivity of the mutant to the control level, measured by 50% infectious dose (ID_50_), indicating that the two virulence factors are interchangeable in this regard. Overproduction of DbpA also allowed the mutant to disseminate to some but not all distal tissues slightly slower than the control, but the mutant with DbpB overproduction showed severely impaired dissemination to all tissues that were analyzed. The mutant with DbpA overproduction colonized all tissues, albeit generating bacterial loads significantly lower than the control in heart and joint, while the mutant overproducing DbpB remained severely defective in heart colonization and registered bacterial loads substantially lower than the control in joint. Taken together, the study indicated that DbpA and DbpB play a similar role in contribution to infectivity as measured by ID_50_ value but contribute differently to dissemination and tissue colonization.

## Introduction

*Borrelia burgdorferi*, the Lyme disease spirochete transmitted by *Ixodes* ticks, causes the most common vector-borne illness in North America and Europe [1], [2]. It is a slow growing bacterium but has a very low 50% infectious dose (ID_50_) in the murine host [3], [4], and can also cause persistent infection despite the development of strong immune responses [5]. Because *B. burgdorferi* is an extracellular pathogen, the interaction with host ligands mediated by spirochetal surface adhesins has been hypothesized to be critical for the pathogenic strategy since early 1990s [6].

*B. burgdorferi* resides primarily in the extracellular matrix (ECM) and connective tissues and between host cells during mammalian infection, where proteoglycans are abundantly found. Pioneering work by Guo *et al.* led to identification of two proteins with approximate molecular masses of 20 kDa on Western blot, which can bind decorin, an important component of proteoglycans; these adhesins were consequently named decorin-binding proteins (Dbps) A and B [6]. Their subsequent study demonstrated that the two adhesins are outer surface lipoproteins and are encoded by a 2-gene operon, in which *dbpA* is located downstream of *dbpB* [7], [8]. At the same time, at least three groups in the field, including Hagman *et al.*, Feng *et al.* and Hanson *et al.*, started exploring the potential of DbpA as a vaccine [8]–[10]. However, the promise quickly diminished after Hagman *et al.* showed that the anti-DbpA response is unable to protect against a challenge via tick infestation, probably because DbpA is not upregulated to a level that can be effectively targeted by antibodies in the tick vector during feeding [11]. A few years later, Leong and colleagues showed that both DbpA and DbpB can also bind glycosaminoglycan, another major component of proteoglycans, albeit exhibiting distinct specificities [12], further strengthening the notion that the adhesins may play a critical role in the pathogenesis of *B. burgdorferi*.

The contribution of DbpA and DbpB to the virulence of *B. burgdorferi* could only be indirectly explored before genetic tools had been available for the pathogen. Earlier studies indicated that mice deficient for decorin become less susceptible to murine Lyme disease and harbor fewer spirochetes during chronic infection [13], [14]. The development in genetic manipulation of *B. burgdorferi* advanced investigation into the role of the *dbpBA* locus to a deeper level. Our recent studies via deletion of the 2-gene operon showed that both DbpA and DbpB are critical for the overall virulence of *B. burgdorferi* [4], [15]. Most recently, Blevins *et al.* reported that at least DbpA is crucial for infectivity [16]. Furthermore, a study through modification of *B. burgdorferi* to increase DbpA expression indicated that higher DbpA expression dramatically enhances the interaction of the pathogen with decorin and, probably as a result, significantly reduces the ID_50_ value and severely impairs dissemination in a murine model [17].

DbpA and DbpB share approximately 40% identity with similar molecular weights, and both bind decorin and glycosaminoglycan and are critical for the overall virulence of *B. burgdorferi* [4], [7]–[9], [12]. In a recent study, we were able to override the essential role of outer surface protein C (OspC) in mammalian infection by modifying an *ospC* mutant to overproduce OspA, OspE, VlsE or DbpA, indicating that OspC functions can be replaced, at least in part, by other outer surface lipoproteins [18]. The *dbpBA* operon is expressed at a moderate level during mammalian infection [17], [19] so their expression can be dramatically increased via genetic manipulation. If increased synthesis of DbpA or DbpB is able to compensate for the deficiency in the other, the study would indicate they are interchangeable. In the current study, to compare the contributions of DbpA and DbpB to the overall virulence, a *dbpBA* mutant was modified to overproduce DbpA or DbpB and examined for the influence on four aspects of the overall virulence, including infectivity, measured by ID_50_ value, pace of dissemination to distal tissues, tissue colonization, measured by the frequency of tissue colonization as well as bacterial load, and persistence in the murine model.

## Results

### Modification of the *dbpAB* mutant to overproduce DbpA or DbpB

**Figure 1:**
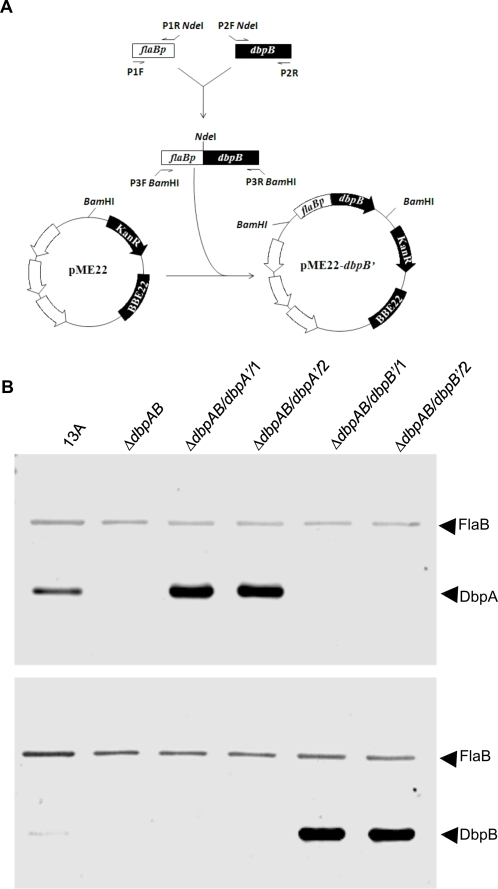
Modification of the *dbpAB* mutant to overproduce DbpA or DbpB. (A) Illustration of pME22-*dbpB'* construction. (B) Confirmation of DbpA and DbpB overproduction by immunoblotting. The parental clone 13A, Δ*dbpAB*, and the clones Δ*dbpAB*/*dbpA'*/1, Δ*dbpAB*/*dbpA'*/2, Δ*dbpAB*/*dbpB'*/1 and Δ*dbpAB*/*dbpB'*/2 were subjected to immunoblot analysis probed with a mixture of FlaB mAb and mouse anti-DbpA (top panel) or -DbpB sera (bottom panel). Lysates prepared from approximately 3×10^6^ organisms were applied to each lane.

The two constructs, pBBE22-*dbpA'* and pME22-*dbpB'*, were electroporated into Δ*dbpAB*, which was generated in our earlier study [4]. pME22-*dbpB'* was constructed from pME22 as illustrated in Figure 1A, while pBBE22-*dbpA'* was modified from pBBE22 in our previous study [17]. The sole difference between pBBE22 and pME22 was the location of the *bbe22* copy; this should not affect the expression activity of the inserted genes. pBBE22-*dbpA'* and pME22-*dbpB'* carried promoterless *dbpA* and *dbpB*, respectively, both of which were fused with a *flaB* promoter. Because Δ*dbpAB* lacks lp25, the plasmid that carries *bbe22* coding for a nicotinamidase essential for survival of *B. burgdorferi* in the mammalian environment [20], both constructs contain a copy of *bbe22*. Fifteen and 23 transformants were obtained from transformation with the constructs. Plasmid analyses led to selection of two clones receiving each construct. These four clones, namely, Δ*dbpAB*/*dbpA'*/1, Δ*dbpAB*/*dbpA'*/2, Δ*dbpAB*/*dbpB'*/1 and Δ*dbpAB*/*dbpB'*/2, shared the same plasmid content as Δ*dbpBA*, which lost cp9, lp5, lp21, lp28-4, lp25 and lp56 [4]. Overproduction of DbpA and DbpB during in vitro growth due to the introduction of the constructs was confirmed by immunoblot analysis (Figure 1B). The DbpA band appeared stronger than DbpB shown for the parental clone 13A; however, this did not necessarily reflect that the 13 spirochetes produced more DbpA than DbpB, because the reactivity of the antisera used in the study might result in different densities of immunoblot bands.

### Either Dbp deficiency or overproduction does not affect spirochete growth in vitro

The influence of Dbp deficiency and overproduction on spirochete growth was investigated. The Δ*dbpAB*, Δ*dbpAB*/*dbpAB*/1, Δ*dbpAB*/*dbpA'*/1 and Δ*dbpAB*/*dbpB'*/1 spirochetes were grown to late-logarithmic (log) phase (∼10^8^ cells/ml), diluted in Barbour-Stoenner-Kelly H (BSK-H) complete medium to a density of ∼10^6^ cells/ml and cultured at 33°C. The bacteria were counted every 12 hours until reaching late-log phase. All the four genotypes completed a generation approximately every 7 hours (data not shown), indicating that either Dbp deficiency or overproduction does not influence in vitro bacterial growth. The morphology and motility of the three genotypes were indistinguishable when assessed under a darkfield microscope.

### Overproduction of DbpA or DbpB dramatically increases binding of specific antibodies to intact spirochetes

Both lipidation and subsequent translocation are posttranslational processes, which determine the cellular location of a lipoprotein [21]. Our modification of *B. burgdorferi* to overproduce DbpA and DbpB did not alter their sequences including signal peptide sequences and thus should not change their cellular locations. Nevertheless, indirect immunofluorescence was used to assess whether the overproduced adhesins were surface exposed. FlaB mAb could not bind its antigen in unfixed spirochetes but resulted in strong fluorescence on all fixed bacteria (Figure S1), indicating that our indirect immunofluoresence procedure did not expose internal antigens of unfixed spirochetes. The *ΔdbpAB* bacteria did not bind either anti-DbpA or -DbpB antibody, while intact *ΔdbpAB*/*dbpAB* organisms showed poor binding. In contrast, modification of the *dbpAB* mutant to overproduce DbpA or DbpB dramatically increased binding of specific anibodies to intact spirochetes, confirming the surface exposure nature of overproduced DbpA and DbpB.

### Overproduction of either DbpA or DbpB reduces the ID_50_ of the *dbpAB* mutant to the control level

Our previous study showed that the lack of either *dbpA* or *dbpB* leads to nearly a 10^3^-fold increase in the ID_50_ value [4]. To investigate whether increasing DbpA or DbpB synthesis can compensate for the deficiency in the other, groups of three BALB/c mice each received one single inoculation of 10^1^ to 10^3^ spirochetes of the clone Δ*dbpAB*/*dbpAB*/1, Δ*dbpAB*/*dbpAB*/2, Δ*dbpAB*/*dbpA'*/1, Δ*dbpAB*/*dbpA'*/2, Δ*dbpAB*/*dbpB'*/1 or Δ*dbpAB*/*dbpB'*/2. The clones Δ*dbpAB*/*dbpAB*/1 and Δ*dbpAB*/*dbpAB*/2, which were used as a control, were generated via introduction of a pBBE22 derivative carrying the *dbpAB* operon in our previous study [17], and thus expressed both *dbpA* and *dbpB* under control of their native promoter. Animals were euthanized 1 month later; heart, joint and skin specimens were subjected to spirochete culture for ID_50_ determination. In two separate experiments, the ID_50_ values for these six clones were determined within a range from 3 to 32 organisms (Table 1). These data indicated that both DbpA and DbpB contribute similarly to the infectivity of *B. burgdorferi*. It also should be pointed out that the genotype Δ*dbpAB*/*dbpAB* registered similar ID_50_ values as the parental clone 13A carrying pBBE22, whose ID_50_ value was measured approximately at 32 organisms [17].

**Table 1:** Overproduction of either DbpA or DbpB reduces the ID_50_ value of the *dbpAB* mutant to the control level.^a^

Expt, clone, dose (no. of organisms)	No. of cultures positive/total no. of specimens examined				No. of mice infected/total no. of mice inoculated	ID_50_ (No. of organisms
	Heart	Joint	Skin	All sites		
I						
Δ*dbpAB*/*dbpAB*/1						6
10^3^	3/3	3/3	3/3	9/9	3/3	
10^2^	3/3	3/3	3/3	9/9	3/3	
10^1^	2/3	2/3	2/3	6/9	2/3	
Δ*dbpAB*/*dbpAB*/2						3
10^3^	3/3	3/3	3/3	9/9	3/3	
10^2^	3/3	3/3	3/3	9/9	3/3	
10^1^	3/3	3/3	3/3	9/9	3/3	
Δ*dbpAB*/*dbpA'*/1						18
10^3^	3/3	3/3	3/3	9/9	3/3	
10^2^	3/3	3/3	3/3	9/9	3/3	
10^1^	0/3	1/3	1/3	2/9	1/3	
Δ*dbpAB*/*dbpA'*/2						6
10^3^	3/3	3/3	3/3	9/9	3/3	
10^2^	3/3	3/3	3/3	9/9	3/3	
10^1^	0/3	1/3	2/3	3/9	2/3	
Δ*dbpAB*/*dbpB'*/1						18
10^3^	0/3	3/3	3/3	6/9	3/3	
10^2^	0/3	3/3	3/3	6/9	3/3	
10^1^	0/3	0/3	1/3	1/9	1/3	
Δ*dbpAB*/*dbpB'*/2						6
10^3^	0/3	3/3	3/3	6/9	3/3	
10^2^	0/3	3/3	3/3	6/9	3/3	
10^1^	0/3	2/3	2/3	4/9	2/3	
II						
Δ*dbpAB*/*dbpAB*/1						18
10^3^	3/3	3/3	3/3	9/9	3/3	
10^2^	3/3	3/3	3/3	9/9	3/3	
10^1^	1/3	1/3	1/3	3/9	1/3	
Δ*dbpAB*/*dbpAB*/2						18
10^3^	3/3	3/3	3/3	9/9	3/3	
10^2^	3/3	3/3	3/3	9/9	3/3	
10^1^	1/3	1/3	1/3	3/9	1/3	
Δ*dbpAB*/*dbpA'*/1						18
10^3^	3/3	3/3	3/3	9/9	3/3	
10^2^	3/3	3/3	3/3	9/9	3/3	
10^1^	0/3	1/3	1/3	2/9	1/3	
Δ*dbpAB*/*dbpA'*/2						18
10^3^	3/3	3/3	3/3	9/9	3/3	
10^2^	3/3	3/3	3/3	9/9	3/3	
10^1^	0/3	1/3	1/3	2/9	1/3	
Δ*dbpAB*/*dbpB'*/1						32
10^3^	0/3	3/3	2/3	5/9	3/3	
10^2^	0/3	1/3	2/3	3/9	2/3	
10^1^	0/3	1/3	1/3	2/9	1/3	
Δ*dbpAB*/*dbpB'*/2						32
10^3^	0/3	3/3	3/3	6/9	3/3	
10^2^	0/3	3/3	3/3	6/9	3/3	
10^1^	0/3	0/3	0/3	0/9	0/3	

a The Δ*dbpAB*/*dbpAB*/1, Δ*dbpAB*/*dbpAB*/2, Δ*dbpAB*/*dbpA'*/1, Δ*dbpAB*/*dbpA'*/2, Δ*dbpAB*/*dbpB'*/1 and Δ*dbpAB*/*dbpB'*/2 spirochetes were grown to late-log phase (10^8^ cells/ml) and 10-fold serially diluted with BSK-H medium. Approximately 100 µl of bacterial suspension was intradermally/subcutaneously inoculated into each BALB/c mouse. Animals were sacrificed 1 month later; heart, tibiotarsal joint and skin specimens were harvested for bacterial isolation. The ID_50_ values were calculated by the method of Reed and Muench [31].

### DbpA is more important than DbpB in the colonization of heart tissue

Our earlier study showed that the deficiency for DbpA alone precludes *B. burgdorferi* from colonizing the heart and that the lack of DbpB leads to a 57% decrease in frequency of heart colonization, but we were not able to conclude whether DbpA is more important than DbpB in the colonization of heart tissue [4]. As shown in Table 1, the *dbpAB* mutant modified with *dbpA* overexpression was recovered from each heart specimen from all of the 24 mice that had received a dose of 10^2^ or 10^3^ organisms, albeit the five infected mice in the lowest dose group did not produce a positive heart specimen. These data indicated that overproducing DbpA alone fully restores the ability of the mutant to colonize the heart, joint and skin tissues. In contrast, although the genotype Δ*dbpAB*/*dbpB'* was recovered from 25 joints and 27 skin specimens of the 27 infected mice, none of the infected mice produced a positive heart culture (Table 1). These results highlight the critical role of DbpA in the colonization of heart tissue.

### Overproduction of either DbpA or DbpB cannot restore the ability of the *dbpAB* mutant to maintain the tissue bacterial load in heart or joint during early infection

**Figure 2:**
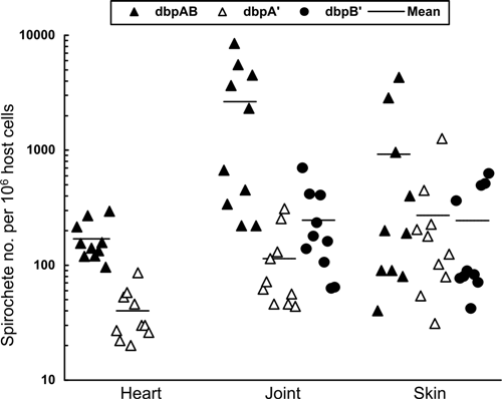
Overproduction of either DbpA or DbpB cannot restore the ability of the *dbpAB* mutant to maintain bacterial loads in heart or joint tissues during early infection. Heart, joint and skin specimens were collected from 30 mice that were infected via a single inoculation with 10^3^ spirochetes of the clone Δ*dbpAB*/*dbpAB*/1, Δ*dbpAB*/*dbpAB*/2, Δ*dbpAB*/*dbpA'*/1, Δ*dbpAB*/*dbpA'*/2, Δ*dbpAB*/*dbpB'*/1 or Δ*dbpAB*/*dbpB'*/2 for 1 month. DNA samples were prepared and analyzed for spirochetal *flaB* and murine actin DNA copies by qPCR. Because the genotype Δ*dbpAB*/*dbpB'* was unable to effectively colonize the heart, the heart specimens of this group were not included. The data are expressed as spirochete numbers per 10^6^ host cells and presented in three groups by combining the subgroups Δ*dbpAB*/*dbpAB*/1 and Δ*dbpAB*/*dbpAB*/2, Δ*dbpAB*/*dbpA'*/1 and Δ*dbpAB*/*dbpA'*/2, and Δ*dbpAB*/*dbpB'*/1 and Δ*dbpAB*/*dbpB'*/2.

The spirochete burden was analyzed to further assess how DbpA and DbpB contribute differently to tissue colonization. Although DbpA overproduction restored the frequency of colonizing the heart tissue, the Δ*dbpAB*/*dbpA'* spirochete load was 4.3-fold lower than that of the genotype Δ*dbpAB*/*dbpAB* (*P* = 1.6×10^−5^) (Figure 2). The Δ*dbpAB*/*dbpAB* bacteria generated loads 23-fold and 11-fold, respectively, higher than those of the Δ*dbpAB*/*dbpA'* (*P* = 0.01) and Δ*dbpAB*/*dbpB'* spirochetes (*P* = 0.02) in the joint. However, all the three genotypes produced similar bacterial loads in skin (*P*<0.05). The two genotypes with adhesin overproduction generated similar bacterial loads in the joint (*P*<0.05). These results indicated that overproduction of either DbpA or DbpB is unable to restore the ability of the *dbpAB* mutant to maintain the bacterial load in either heart or joint tissue.

### Overproduction of DbpA makes the *dbpAB* mutant disseminate more efficiently to distal tissues than increased DbpB production

Dissemination was assessed as another aspect of the overall virulence. Groups of six to 21 BALB/c mice each received a single intradermal/subcutaneous inoculation of 10^5^ spirochetes of the clone Δ*dbpAB*/*dbpAB*/1, Δ*dbpAB*/*dbpAB*/2, Δ*dbpAB*/*dbpA'*/1, Δ*dbpAB*/*dbpA'*/2, Δ*dbpAB*/*dbpB'*/1 or Δ*dbpAB*/*dbpB'*/2. This dose, 1000-fold higher than the ID_50_ value, was used to ensure that all inoculated mice were infected. Three animals from each group were euthanized at 1-week intervals; inoculation site and remote site skin, heart and joint specimens were harvested for spirochete isolation. Bacteria were injected into the dermis of the chest so skin specimens from the back were harvested as remote sites. As a positive control, the Δ*dbpAB*/*dbpAB*/1 and Δ*dbpAB*/*dbpAB*/2 bacteria were grown from 6 joint, 5 remote skin, and 2 heart specimens of 6 examined mice at 1 week; all sites became culture-positive at 2 weeks after inoculation (Table 2), consistent with our previous study that showed the parental clone 13A carrying pBBE22 colonized these tissues within 2 weeks [22]. The Δ*dbpAB*/*dbpA'*/1 and Δ*dbpAB*/*dbpA'*/2 bacteria were grown from 6 joint, 4 remote skin and 2 heart specimens of 6 examined mice at 1 week, and from 6 joint, 5 remote skin and 3 heart specimens of 6 mice at 2 weeks; all sites became culture-positive at 3 weeks. These results indicated that DbpA overproduction allowed the mutant to disseminate to the distal tissues that were analyzed slightly slower than the mutant restored with *dbpAB* expression. In contrast, the mutant overproducing DbpB disseminated extremely slowly; the first positive remote skin and heart specimens were not detected until 3 and 6 weeks, respectively. Only one out of the six remote skin specimens produced a positive culture at 4 weeks. It should be pointed out that the 10^5^-dose inoculation apparently caused delayed dissemination, as nearly all skin samples were culture-positive when the dose of 10^2^ to 10^3^ organisms was used to determine the ID_50_ value for the genotype Δ*dbpAB*/*dbpB'* (Table 1). The high-dose inoculation might induce a faster and stronger immune response, which slowed the dissemination process, when the strain had a severe defect in dissemination. This was also noted in our previous study [17].

**Table 2:** The *dbpAB* mutant with DbpA overproduction disseminates to distal tissues more efficiently than the mutant overproducing DbpB.^a^

Clone	No. of specimens positive/total no. of specimens examined at post-inoculation weeks															
	1				2				3				4			
	I.S.	R.S.	Heart	Joint	I.S.	R.S.	Heart	Joint	I.S.	R.S.	Heart	Joint	I.S.	R.S.	Heart	Joint
Δ*dbpAB*/*dbpAB*/1	3/3	2/3	1/3	3/3	3/3	3/3	3/3	3/3	ND^b^	ND	ND	ND	ND	ND	ND	ND
Δ*dbpAB*/*dbpAB*/2	3/3	3/3	1/3	3/3	3/3	3/3	3/3	3/3	ND	ND	ND	ND	ND	ND	ND	ND
Δ*dbpAB*/*dbpA'*/1	3/3	2/3	1/3	3/3	3/3	3/3	1/3	3/3	3/3	3/3	3/3	3/3	ND	ND	ND	ND
Δ*dbpAB*/*dbpA'*/2	3/3	2/3	1/3	3/3	3/3	2/3	2/3	3/3	3/3	3/3	3/3	3/3	ND	ND	ND	ND
Δ*dbpAB*/*dbpB'*/1	ND	ND	ND	ND	3/3	0/3	0/3	3/3	3/3	1/3	0/3	3/3	3/3	1/3	0/3	3/3
Δ*dbpAB*/*dbpB'*/2	ND	ND	ND	ND	3/3	0/3	0/3	2/3	3/3	0/3	0/3	2/3	2/3	0/3	0/3	2/3

Clone	No. of specimens positive/total no. of specimens examined at post-inoculation weeks											
	5				6				7			
	I.S.	R.S.	Heart	Joint	I.S.	R.S.	Heart	Joint	I.S.	R.S.	Heart	Joint
Δ*dbpAB*/*dbpAB*/1	ND	ND	ND	ND	ND	ND	ND	ND	ND	ND	ND	ND
Δ*dbpAB*/*dbpAB*/2	ND	ND	ND	ND	ND	ND	ND	ND	ND	ND	ND	ND
Δ*dbpAB*/*dbpA'*/1	ND	ND	ND	ND	ND	ND	ND	ND	ND	ND	ND	ND
Δ*dbpAB*/*dbpA'*/2	ND	ND	ND	ND	ND	ND	ND	ND	ND	ND	ND	ND
Δ*dbpAB*/*dbpB'*/1	3/3	3/3	0/3	3/3	3/3	3/3	1/3	3/3	2/3	2/3	2/3	2/3
Δ*dbpAB*/*dbpB'*/2	3/3	2/3	0/3	3/3	2/3	2/3	0/3	2/3	3/3	2/3	1/3	2/3

a Groups of 6–21 BALB/c mice each received a single intradermal/subcutaneous injection of 10^5^ organisms of the clone Δ*dbpAB*/*dbpAB*/1, Δ*dbpAB*/*dbpAB*/2, Δ*dbpAB*/*dbpA'*/1, Δ*dbpAB*/*dbpA'*/2, Δ*dbpAB*/*dbpB'*/1 or Δ*dbpAB*/*dbpB'*/2. Three animals from each group were euthanized at 1-week intervals; inoculation site (I.S.) and remote site (R.S.) skin, heart, and joint specimens were harvested for spirochete isolation. The I.S. site was at the chest; therefore the R.S. site was at the back of mice.

b ND, not determined.

We previously showed that modification of *B. burgdorferi* to overproduce DbpA results in severely impaired dissemination based on an ear biopsy study [17]. Our subsequent studies showed that ear is the last tissue to be colonized by *B. burgdorferi* during murine infection [18]. To investigate how slowly the mutant overproducing the adhesins disseminated to ear, subgroups of three BALB/c mice each received one single inoculation of 10^5^ spirochetes of the clone Δ*dbpAB*/*dbpAB*/1, Δ*dbpAB*/*dbpAB*/2, Δ*dbpAB*/*dbpA'*/1, Δ*dbpAB*/*dbpA'*/2, Δ*dbpAB*/*dbpB'*/1 or Δ*dbpAB*/*dbpB'*/2. Ear biopsies were taken for bacterial culture every week up to 7 weeks post-inoculation. At 2 weeks post-inoculation, all of the six mice inoculated with the genotype Δ*dbpAB*/*dbpAB* had a positive biopsy (data not shown). In contrast, none of the mice inoculated with the genotype Δ*dbpAB*/*dbpA'* produced a positive biopsy within the first 3 weeks but all of the six gave a positive result at 4 weeks. The six mice receiving the Δ*dbpAB*/*dbpB'* did not produce a positive ear biopsy until week 6 or 7. The study indicated that the genotype Δ*dbpAB*/*dbpA'* disseminates to the ear tissue at a much slower pace than the control but much faster than the genotype Δ*dbpAB*/*dbpB'*. The study again highlights the difference of the adhesins to influence the dissemination of *B. burgdorferi*.

### Strong specific humoral responses are unable to effectively target *B. burgdorferi* overproducing DbpA or DbpB in chronically infected mice

Subgroups of five BALB/c mice each received one single intradermal/subcutaneous injection of 10^5^ spirochetes of the clone Δ*dbpAB*/*dbpAB*/1, Δ*dbpAB*/*dbpAB*/2, Δ*dbpAB*/*dbpA'*/1, Δ*dbpAB*/*dbpA'*/2, Δ*dbpAB*/*dbpB'*/1 or Δ*dbpAB*/*dbpB'*/2. One of the five mice that were challenged with the clone Δ*dbpAB*/*dbpB'*/1 was lost during the study. The remaining 29 animals were euthanized 4 months post-inoculation. *B. burgdorferi* was grown from each skin, heart and joint sample from all of the 10 mice that had been challenged with the clone Δ*dbpAB*/*dbpAB*/1 or Δ*dbpAB*/*dbpAB*/2 (Table 3). Similarly, the Δ*dbpAB*/*dbpA'*/1 and Δ*dbpAB*/*dbpA'*/2 spirochetes were recovered from 9 of the 10 heart specimens and all of the joint and skin samples of the 10 infected mice, and the Δ*dbpAB*/*dbpB'*/1 and Δ*dbpAB*/*dbpB'*/2 spirochetes were grown from all joint specimens, 8 of 9 skin samples and 2 of 9 hearts. Again, the study indicated that DbpA is more important than DbpB in contributing to the colonization of heart.

**Table 3:** The *dbpAB* mutant overeproducing DbpA or DbpB causes persistent infection.^a^

Clone	No. of cultures positive/total no. of specimens examined				No. of mice infected/total no. of mice inoculated
	Heart	Joint	Skin	All sites	
Δ*dbpAB*/*dbpAB*/1	5/5	5/5	5/5	15/15	5/5
Δ*dbpAB*/*dbpAB*/2	5/5	5/5	5/5	15/15	5/5
Δ*dbpAB*/*dbpA'*/1	4/5	5/5	5/5	14/15	5/5
Δ*dbpAB*/*dbpA'*/2	5/5	5/5	5/5	15/15	5/5
Δ*dbpAB*/*dbpB'*/1	0/4	4/4	3/4	7/12	4/4
Δ*dbpAB*/*dbpB'*/2	2/5	5/5	5/5	12/15	5/5

Groups of five BALB/c mice were inoculated with the clone Δ*dbpAB/dbpAB*/1, Δ*dbpAB/dbpAB*/2, Δ*dbpAB/dbpA'*/1, Δ*dbpAB/dbpA'*/2, Δ*dbpAB/dbpB'*/1 or Δ*dbpAB/dbpB'*/2 and sacrificed 4 months later. One animal that was challenged with the clone Δ*dbpAB/dbpB'*/1 was lost during the study. Heart, tibiotarsal joint and skin specimens were harvested for spirochete culture.

**Figure 3:**
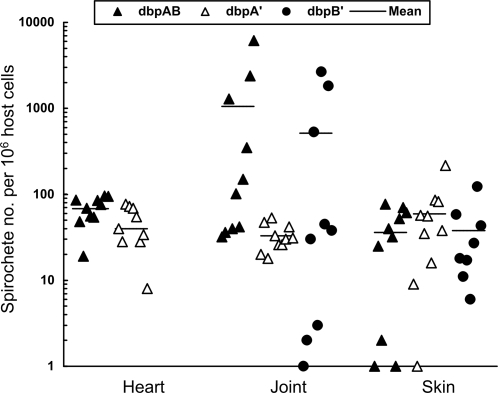
Strong humoral responses cannot effectively target the *dbpAB* mutant overproducing DbpA or DbpB in chronically infected mice. Subgroups of four or five BALB/c mice each received one single intradermal/subcutaneous injection of 10^5^ spirochetes of the clone Δ*dbpAB*/*dbpAB*/1, Δ*dbpAB*/*dbpAB*/2, Δ*dbpAB*/*dbpA'*/1, Δ*dbpAB*/*dbpA'*/2, Δ*dbpAB*/*dbpB'*/1 or Δ*dbpAB*/*dbpB'*/2, and euthanized 4 months later. DNA samples were prepared from samples with a positive culture and analyzed for spirochetal *flaB* and murine actin DNA copies by qPCR. The data are expressed as spirochete numbers per 10^6^ host cells and presented in three groups by combining the subgroups Δ*dbpAB*/*dbpAB*/1 and Δ*dbpAB*/*dbpAB*/2, Δ*dbpAB*/*dbpA'*/1 and Δ*dbpAB*/*dbpA'*/2, and Δ*dbpAB*/*dbpB'*/1 and Δ*dbpAB*/*dbpB'*/2.

The spirochetal burden was analyzed to further assess whether overproduction of the adhesins affected persistence during chronic infection. A total of 78 culture-positive heart, joint and skin specimens from the 29 infected mice were processed for DNA and analyzed for the bacterial load. Although both culture-positive heart specimens from the two mice infected with the clone Δ*dbpAB*/*dbpB'*/1 contained a very low bacterial load, they were not shown because of the small sample size. The genotype Δ*dbpAB*/*dbpA'* registered a spirochete load approximately 23% lower than the genotype Δ*dbpAB*/*dbpAB* in the heart tissue (*P* = 0.02) (Figure 3). However, all the three genotypes generated similar bacterial loads in either joint (*P*<0.05) or skin tissue (*P*<0.05), indicating that the mutant overproducing either DbpA or DbpB is not more effectively targeted than the control by humoral responses induced during chronic murine infection.

*B. burgdorferi* recovered from all the 48 positive heart, joint, and skin specimens of the 19 mice infected with the clone Δ*dbpAB*/*dbpA'*/1, Δ*dbpAB*/*dbpA'*/2, Δ*dbpAB*/*dbpB'*/1 or Δ*dbpAB*/*dbpB'*/2 was analyzed for possible mutations. Isolates were grown to late-log phase and examined for DbpA and DbpB synthesis using immunoblotting. The purpose of this experiment was two-fold. First, if the shuttle vector was lost, its carried *dbpA* or *dbpB* gene would be lost and consequently no DbpA or DbpB band should be detectable. Second, if deletion or nonsense mutation occurred on the *dbpA* or *dbpB* copy, no DbpA or DbpB, or only truncated versions would be detected on immunoblots. All 48 isolates abundantly produced either DbpA or DbpB depending on the constructs they received (data not shown), indicating that the unlikelihood of mutations occurred on the introduced *dbpA* or *dbpB* copy and that the constructs were well maintained during chronic infection.

**Figure 4:**
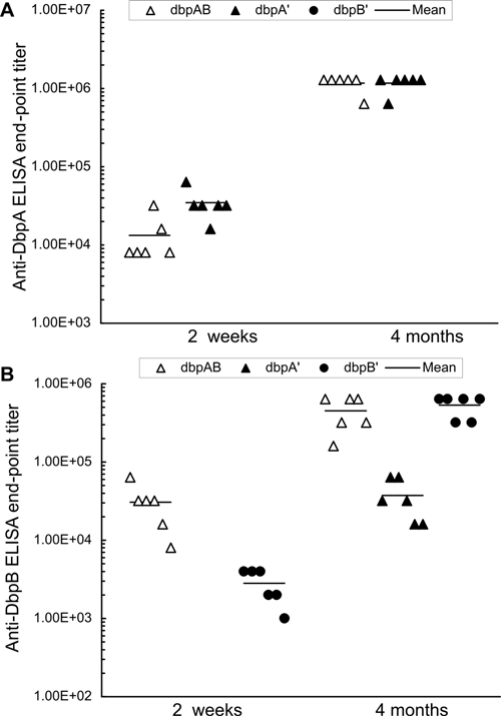
Anti-DbpA and -DbpB humoral responses were induced during acute and chronic infection. Sera were collected from the 18 mice that received one single intradermal/subcutaneous injection of 10^5^ spirochetes of the clone Δ*dbpAB*/*dbpAB*/1, Δ*dbpAB*/*dbpAB*/2, Δ*dbpAB*/*dbpA'*/1, Δ*dbpAB*/*dbpA'*/2, Δ*dbpAB*/*dbpB'*/1 or Δ*dbpAB*/*dbpB'*/2, and euthanized at 2 weeks. An additional 18 samples were randomly chosen from the 29 mice that were infected for 4 months. Anti-DbpA (A) and -DbpB titers (B) were determined by end-point ELISAs.

Next, the humoral responses to DbpA and DbpB during acute (2 weeks) and chronic infection (4 months) were analyzed by ELISAs. Serum samples were collected from the 18 mice that were euthanized at 2 weeks post-inoculation and had been used to generate data for Table 2. An additional 18 sera were randomly selected from the 29 blood samples that were collected at 4 months post-inoculation from the 29 mice, which had been used to generate data for Table 3. As expected, no anti-DbpA response was detected in the 12 samples from mice that were infected with the genotype Δ*dbpAB*/*dbpB'* either for 2 weeks or 4 months (Figure 4A). Infection with the genotype Δ*dbpAB*/*dbpA'* elicited an anti-DbpA response 2.6-fold stronger than inoculation with the Δ*dbpAB*/*dbpAB* spirochetes at 2 weeks (*P* = 0.02), but both phenotypes induced similar responses at 4 months (*P* = 1.0), indicating that increased DbpA synthesis triggers a faster humoral response. Although the genotype Δ*dbpAB*/*dbpA'* did not induce a detectable anti-DbpB response at 2 weeks, it did elicit a significant response during chronic infection (Figure 4B). In spite of DbpB overproduction, the Δ*dbpAB*/*dbpB'* spirochetes elicited an anti-DbpB response 11-fold lower than the genotype Δ*dbpAB*/*dbpAB* at 2 weeks (*P* = 0.005), but both phenotypes induced similar responses during chronic infection (*P* = 0.48).

## Discussion

As an extracellular bacterium, *B. burgdorferi* resides primarily in the ECM and connective tissues, and between host cells during mammalian infection, where host ligands for both DbpA and DbpB, including decorin and glycosaminoglycan, are abundantly present. Thus, the interplay of the pathogen with these host ligands, mediated by the adhesins, may affect various aspects of its overall virulence [23], [24]. Our previous studies showed that neither adhesin is essential for infection but both are critical for the overall virulence [4], [15]. The current study focused on comparing differences between the adhesins in contribution to the overall virulence. We showed that overproduction of either adhesin fully compensates for the lack of the other in the restoration of infectivity, as defined by ID_50_ value, indicating that both adhesins contribute similarly to this aspect of virulence. However, overproduction of either was unable to fully restore the ability of the *dbpAB* to disseminate to distal tissues or to maintain the bacterial load during early murine infection. Therefore, the study also highlighted that the adhesins contribute differently to the overall virulence of *B. burgdorferi*.

Our previous study showed that the lack of either DbpA or DbpB results in nearly a 10^3^-fold decrease in infectivity [4]. Infectivity, measured by ID_50_ value, was assessed as the first aspect of the overall virulence in the current study. Overproduction of either DbpA or DbpB fully restored the *dbpAB* mutant with infectivity to the control level, indicating that either virulence factor is able to replace the other when produced at high levels. This finding is crucial for the subsequent evaluation of other aspects of the overall virulence, because the ID_50_ value is the best reflection for the ability of a pathogen to evade the initial elimination by the first line of host defense. Only when the three genotypes were equally well protected against immune defenses, the comparison in other aspects, such as dissemination and tissue colonization, would be meaningful.

After initial replication at the inoculation site, *B. burgdorferi* disseminates to distal tissues. This ability was evaluated as a second aspect of the overall virulence. The *dbpAB* mutant with DbpA overproduction disseminated to each type of the distal tissues that were analyzed at a much faster pace than that with DbpB overproduction, highlighting the difference of the adhesins. Although DbpA overproduction restored the ability of the mutant to disseminate to joint, heart and remote skin tissues nearly to the control level, its dissemination to ear remained severely impaired. Our current and previous studies consistently showed an order of tissue colonization by *B. burgdorferi*, as the joint is first colonized, followed by heart and remote skin, while ear is always the last [18]. This sequential tissue colonization raises a question about how *B. burgdorferi* disseminates to distal tissues. If it disseminates via the bloodstream, how could the ear be colonized so late in some cases? While it remains to be addressed how *B. burgdorferi* disseminates, our studies clearly showed a great influence of the expression levels of the adhesins on this event. Our previous study indicated that increasing production of DbpA, although reducing the ID_50_ value, severely impairs the dissemination to ear tissue [17]. Our most recent study showed that increasing DbpA synthesis is able to protect an *ospC* mutant in immunodeficient mice but is unable to restore the mutant with the ability to cause disseminated infection [18]. Taken together, these studies indicate that the DbpA production level significantly influences the dissemination of *B. burgdorferi* and that this influence is also largely affected by the presence of other surface lipoproteins.

The slow dissemination of the genotype Δ*dbpAB*/*dbpB'* is also indirectly reflected by a weak anti-DbpB response induced during early infection. The severely impaired dissemination may limit a quick expansion of the total bacterial load and thus affects the immune response. This slow expansion could be a reason why infection with the genotype Δ*dbpAB*/*dbpB'* did not elicit a humoral response cross-reactive with DbpA, although the Δ*dbpAB*/*dbpA'* spirochetes stimulated a significant response cross-reactive with DbpB during chronic infection. However, our study could not rule out that DbpB may be less immunogenic than DbpA during borrelial infection. Nevertheless, the antigenic cross-reactivity between the adhesins had never been reported.

The ability to colonize tissues was assessed as a third aspect of the overall virulence. DbpA overproduction restored this ability of the *dbpAB* mutant to the control level, if measured by frequency of tissue colonization. When the bacterial load was analyzed, however, our data indicated that the overproduction far less restored the ability to maintain the bacterial loads either in heart or joint tissue during acute infection. In contrast, the mutant with DbpB overproduction was not recovered from any of the heart specimens within the first 5 weeks post-inoculation, but it was indeed grown from some heart specimens between 6 weeks and 4 months of infection. Slower dissemination could be a contributing factor for the negative heart culture, when bacterial isolation was conducted within 1 month post-inoculation. However, the study clearly showed that overproduction of DbpA, more effectively than that of DbpB, restores the colonization of heart tissue. In joint and skin tissues, overproduction of either adhesin had similar effects on this aspect of virulence.

Persistence was examined as a fourth aspect of the overall virulence. Overproduction of either adhesin did not reduce the ability to cause chronic infection, as measured by bacterial load. During early infection (1 month), the Δ*dbpAB*/*dbpA'* load was 4.3-fold lower than that of the genotype Δ*dbpAB*/*dbpAB* in heart; the difference reduced to 1.7-fold after 4 months of infection. A more dramatic change was noted in joint tissue, where the Δ*dbpAB*/*dbpAB* load was 23-fold and 11-fold, respectively, higher than those of the genotypes Δ*dbpAB*/*dbpA'* and Δ*dbpAB*/*dbpB'* at 1 month, but the differences became indistinguishable among the three genotypes after 4 months of infection. In spite of robust anti-DbpA and -DbpB humoral responses, the mutant overproducing either adhesin persisted as well as the control. This is unexpected because our previous study showed that overproduction of DbpA diminishes the ability of *B. burgdorferi* to persist in heart and joint tissues of chronically infected immunocompetent mice [17], although specific DbpA antibody is unable to reduce the bacterial load of wild-type *B. burgdorferi* [25]. Two obvious differences can be used to interpret these observations. First, in the previous study the parental strain produces both DbpA and DbpB from the native *dbpBA* locus, so further introduced DbpA overproduction may dramatically increase accumulation of the adhesins on the spirochete's surface and, as a consequence, may improve the effectiveness of specific antibodies to target *B. burgdorferi* in heart and joint tissues. Second, because the genotypes Δ*dbpAB*/*dbpA'* and Δ*dbpAB*/*dbpB*' lack either DbpA or DbpB, the deficiency for either may make specific antibodies to the other less effective in killing the pathogen or in other words, the anti-DbpA and -DbpB responses may have a synergetic effect in controlling *B. burgdorferi*.

A recent study by Blevins *et al.* reported that DbpA but not DbpB is critical for infectivity measured by the ID_50_ value [16], while our previous study showed that both adhesins are crucial to this aspect of overall virulence [4]. The intrinsic difference of the bacterial strains (297 used in their study vs. B31 13A in ours) could cause the disparity. For instance, if the native promoter of the *dbpBA* operon of the strain 297 drives more active expression than that of B31, the *dbpA* gene alone controlled by the *dbpBA* promoter may sufficiently restore the *dbpAB* mutant with full infectivity, as the current study showed that both DbpA and DbpB are interchangeable in contribution to infectivity as measured by ID_50_ value when produced at a high level. They also reported that their *dbpAB* mutant becomes less impaired following tick challenge. In response to a fresh bloodmeal *B. burgdorferi* undergoes a dramatic change, in terms of gene expression, a process that prepares the pathogen for mammalian infection. This adaptation process should certainly be considered as a contributing factor to increase infectivity of their *dbpAB* mutant. The fact that ticks acquired the *dbpAB* mutant organisms from infected animals must also be considered. The genetic manipulation of *B. burgdorferi* is time-consuming, and it often takes a long period of time to generate, characterize and select a mutant. These processes may attenuate the infectivity of generated mutants, as it has been known for more than two decades that prolonged in vitro cultivation reduces the infectivity of *B. burgdorferi* [26]. We noticed that several of our genetically modified strains, including the *dbpAB* mutant, showed improvement in infectivity when isolates that were recovered from infected mice were used as an inoculum (Xu and Liang, unpublished data).

Although both DbpA and DbpB are believed to be well characterized outer surface lipoproteins [7]–[9], our immunofluorescence showed limited surface exposure of the lipoproteins on spirochetes grown in vitro. This observation is consistent with a study by Radolf and colleagues, which showed limited surface exposure of OspA, OspB and OspC on cultured *B. burgdorferi* by immunofluorescence [27]. However, our finding that limited exposure of DbpA and DbpB on the surface of cultured spirochetes does not suggest that the adhesins can not have a full access to their ligand decorin in mammalian tissues because OspA and OspB, which are abundantly produced in spirochetes grown in vitro but are downregulated to a baseline level during mammalian infection [28], may reduce the accessibility of DbpA and DbpB. Our modification of *B. burgdorferi* to overproduce DbpA and DbpB led to a dramatically increased binding of the specific antibodies to intact spirochetes as showed by immunofluorescence, indicating that both adhesins are surface exposed.

Both DbpA and DbpB are surface lipoprotein adhesins with similar molecular weights and encoded by the same operon, share approximately 40% identity, and bind host decorin and glycosaminoglycans [7]–[9], [12]. The current study revealed that the adhesins also share cross-antigenic reactivity. These similarities can be used to explain why the two virulence factors can be interchangeable in the protection of *B. burgdorferi* against initial elimination by immune defenses when produced at an increased level; however, they indeed contribute differently to dissemination and tissue colonization.

## Materials and Methods

### Strains and constructs generated previously but used in the current study

The *B. burgdorferi* B31 clone 13A, the *dbpAB* mutant (Δ*dbpAB*) and the complemented clones Δ*dbpAB/dbpAB*/1 and Δ*dbpAB/dbpAB*/2, the shuttle vector pME22, and the recombinant plasmids pME22-*dbpB* and pBBE22-*dbpA'* were generated in our previous studies [4], [17]. The features of these clones and constructs were summarized in Table 4.

**Table 4:** Constructs and clones used in the study.

Construct or clone	Description	Source
pME22	pBSV2 carrying a *bbe22* copy	Reference [4]
pME22-*dbpB*	pME22 carrying a *dbpB* copy	Reference [4]
pBBE22-*dbpA'*	pBBE22 carrying promoterless *dbpA* fused with *flaB* promoter	Reference [17]
pME22-*dbpB'*	pME22 carrying promoterless *dbpB* fused with *flaB* promoter	This study
13A	Cloned from *B. burgdorferi* B31 A13	Reference [33]
Δ*dbpAB*	*dbpAB* mutant	Reference [4]
Δ*dbpAB*/*dbpAB*/1	Expressing *dbpA* and *dbpB* driven by their own promoter	Reference [4]
Δ*dbpAB*/*dbpAB*/2	Expressing *dbpA* and *dbpB* driven by their own promoter	Reference [4]
Δ*dbpAB*/*dbpA'*/1	Expressing *dbpA* driven by *flaB* promoter	This study
Δ*dbpAB*/*dbpA'*/2	Expressing *dbpA* driven by *flaB* promoter	This study
Δ*dbpAB*/*dbpB'*/1	Expressing *dbpB* driven by *flaB* promoter	This study
Δ*dbpAB*/*dbpB'*/2	Expressing *dbpB* driven by *flaB* promoter	This study

### Construction of pBBE22-*dbpB'*

As illustrated in Figure 1A, a 258-bp fragment of the *flaB* promoter region was amplified with use of the primers P1F and P1R. The sequences and restriction enzyme sites of the primers used in the study were presented in Table 5. A 1152-bp fragment covering the entire coding region of the *dbpB* gene and some sequences from the construct pME22-*dbpB*, which was used as a template, was amplified with use of the primers P2F and P2R. The two PCR products were pooled, purified by using the QIAquick PCR purification kit (QIAGEN Inc., Valencia, CA), digested with *Nde*I, repurified, and ligated. The resultant product was used as a template and amplified by nested PCR with use of the primers P3F and P3R. The PCR product was purified, digested with *Bam*HI, and cloned into the shuttle vector pME22 [4]. The insert and flanking regions within the recombinant plasmid were sequenced to ensure the construct was as designed.

**Table 5:** Primers used in the study.

Primer	Sequence (5′ to 3′)^a^
P1F	AGAAGTACGAAGATAGAGAGAGAAA
P1R	AACACATATGTCATTCCTCCATGATAAA
P2F	AACATATGAAAATTGGAAAGCTAAATTCA
P2R	GGAAATCTTCCTTGAAGCT
P3F	ATAGGATCCAAGATAGAGAGAGAAAAGT
P3R	TTGGATCCTGATTATCGGGCGAAGAG

The underlined sequences are restriction enzyme sites: *Nde*I sites (P1R and P2F), and *Bam*HI sites (P3F and P3R).

### Modification of the *dbpAB* mutant to overproduce DbpA or DbpB

pBBE22-*dbpA'* and pME22-*dbpB'* were electroporated into Δ*dbpAB*; resulting transformants were screened as described previously [4]. Identified transformants were first surveyed for the presence of lp28-1 because this plasmid is essential for persistent infection of immunocompetent hosts [20], [29]. Only clones containing lp28-1 were further analyzed for plasmid content as described previously [30]. Overexpression of DbpA and DbpB was verified by using immunoblot analysis probed with a mixture of a FlaB mAb and anti-DbpA or -DbpB sera as described in an earlier study [15].

### In vitro growth study

Spirochetes were grown to late-log phase (∼10^8^ cells per ml), diluted in BSK-H complete medium (Sigma Chemical Co., St. Louis, MO) to a density of ∼10^6^ cells per ml and cultured at 33°C. The bacteria were counted every 12 hours until they reached late-log phase.

### Indirect immunofluorescence

Indirect immunofluorescence was performed on both unfixed and fixed spirochetes. Briefly, *B. burgdorferi* was grown to late-log phase (10^8^ cells/ml) in BSK-H complete medium at 33°C. For fluorescent labeling of unfixed spirochetes, approximately 2×10^7^ cells were harvested from 0.2 ml of culture by centrifugation at 4,000×*g* for 20 min, gently suspended in 100 µl PBS supplemented with 2 µl of mouse anti-DbpA or -DbpB sera, or FlaB mAb preparation, and incubated for 1 hour at room temperature. After 2 washes with excess volumes of PBS by centrifugation at 16,000×*g* for 5 min, spirochetes were resuspended in 100 µl of PBS containing 1.0 µg of fluorescein isothiocyanate-conjugated goat anti-mouse IgG (Pierce Chemical Company, Rockford, IL), incubated for 1 hour, washed twice with PBS by centrifugation, resuspended in 50 µl PBS, applied to microscopic slides, and analyzed using Axio Imager (Carl Zeiss Microimaging, Inc., Thornwood, NY).

For fluorescent labeling of fixed spirochetes, organisms were harvested from 0.2 ml of culture by centrifugation, suspended in 50 µl of PBS, followed by adding 1.0 ml of acetone, incubated at room temperature for 20 min, and then centrifuged at 16,000×*g* for 5 min. After being washed once with PBS, fixed spirochetes were processed as described above for fluorescent labeling of unfixed bacteria.

### Determination of ID_50_ values

The ID_50_ values were determined in two separate experiments. In each experiment, spirochetes were grown to late-log phase (10^8^ cells per ml) in BSK-H medium at 33°C and 10-fold serially diluted with the medium. BALB/c mice (age, 4 to 6 weeks; provided by the Division of Laboratory Animal Medicine at Louisiana State University, Baton Rouge, LA) each received one single intradermal/subcutaneous injection of 100 µl of spirochetal suspension. Mice were euthanized 1 month post-inoculation; heart, tibiotarsal joint and skin (not from inoculation site) specimens were harvested for bacterial culture and DNA preparation as described previously [30]. The ID_50_ value was calculated as described by Reed and Muench [31]. DNA was extracted from selective specimens and used for analysis of tissue bacterial loads as described below. All animal procedures described here and below were approved by the Institutional Animal Care and Use Committee at Louisiana State University.

### Quantification of tissue spirochetal load

DNA was extracted for heart, joint and skin specimens and quantified for the copy numbers of *flaB* and murine actin genes by quantitative PCR (qPCR) as previously described [30]. The tissue spirochete burden was expressed as *flaB* DNA copies per 10^6^ host cells (2×10^6^ actin DNA copies).

### Dissemination and chronic infectivity studies

BALB/c mice each received one single intradermal/subcutaneous injection of 10^5^ spirochetes. In a dissemination study, inoculated mice were euthanized at 1-week intervals for up to 7 weeks, starting at 1 week; inoculation site and remote skin, heart and joint specimens were aseptically harvested for spirochete isolation as previously described [30]. Because spirochetes were injected into the chest skin, the back skin was harvested as a remote site. In a second dissemination study, inoculated mice were subjected to ear biopsy for up to 7 weeks as described previously [17]. In a chronic study, inoculated animals were euthanized 4 months post-inoculation; heart, tibiotarsal joint and skin specimens were aseptically collected for spirochete culture and DNA extraction. DNA was prepared for analysis of tissue bacterial loads as described above. Serum samples were collected and stored in case an immune response analysis was needed.

### Mutation analysis

Spirochetes were isolated from chronically infected mice, grown to late-log phase in BSK-H complete medium, harvested by centrifugation and analyzed for DbpA and DbpB synthesis by immunoblot analysis probed with a mixture of a FlaB mAb and anti-DbpA or -DbpB sera as described above.

### End-point ELISA titers

Specific DbpA and DbpB antibody end-point titers were determined by ELISAs. Ninety-six-well plates (Fisher Scientific, Pittsburgh, PA) were coated with 100 µl of 2.0 µg/ml recombinant DbpA or DbpB per well. The recombinant proteins were prepared as described in our earlier study [15]. Sera were two-fold serially diluted, starting at 1∶1000. Five samples drawn from naive BALB/c mice were used as a control. The ELISA was performed as previously described [32].

### Statistical analysis

A one-way analysis of variance (ANOVA) was used to analyze data, followed by a two-tailed Student *t* test to calculate a *P* value for each two groups. A *P* value≤0.05 was considered to be significant.

## Supporting Information

Figure S1 (13.57 MB PDF)

## References

[1] Burgdorfer W, Hayes SF, Benach JL (1988). Development of *Borrelia burgdorferi* in ixodid tick vectors.. Ann N Y Acad Sci.

[2] Steere AC (2001). Lyme disease.. N Engl J Med.

[3] Barthold SW (1991). Infectivity of *Borrelia burgdorferi* relative to route of inoculation and genotype in laboratory mice.. J Infect Dis.

[4] Shi Y, Xu Q, McShan K, Liang FT (2008). Both decorin-binding proteins A and B are critical for the overall virulence of *Borrelia burgdorferi*.. Infect Immun.

[5] Seiler KP, Weis JJ (1996). Immunity to Lyme disease: protection, pathology and persistence.. Curr Opin Immunol.

[6] Guo BP, Norris SJ, Rosenberg LC, Hőők M (1995). Adherence of *Borrelia burgdorferi* to the proteoglycan decorin.. Infect Immun.

[7] Guo BP, Brown EL, Dorward DW, Rosenberg LC, Hőők M (1998). Decorin-binding adhesins from *Borrelia burgdorferi*.. Mol Microbiol.

[8] Hagman KE, Lahdenne P, Popova TG, Porcella SF, Akins DR (1998). Decorin-binding protein of *Borrelia burgdorferi* is encoded within a two-gene operon and is protective in the murine model of Lyme borreliosis.. Infect Immun.

[9] Feng S, Hodzic E, Stevenson B, Barthold SW (1998). Humoral immunity to *Borrelia burgdorferi* N40 decorin binding proteins during infection of laboratory mice.. Infect Immun.

[10] Hanson MS, Cassatt DR, Guo BP, Patel NK, McCarthy MP (1998). Active and passive immunity against *Borrelia burgdorferi* decorin binding protein A (DbpA) protects against infection.. Infect Immun.

[11] Hagman KE, Yang X, Wikel SK, Schoeler GB, Caimano MJ (2000). Decorin-binding protein A (DbpA) of *Borrelia burgdorferi* is not protective when immunized mice are challenged via tick infestation and correlates with the lack of DbpA expression by *B. burgdorferi* in ticks.. Infect Immun.

[12] Fischer JR, Parveen N, Magoun L, Leong JM (2003). Decorin-binding proteins A and B confer distinct mammalian cell type-specific attachment by *Borrelia burgdorferi*, the Lyme disease spirochete.. Proc Natl Acad Sci U S A.

[13] Liang FT, Brown EL, Wang T, Iozzo RV, Fikrig E (2004). Protective niche for *Borrelia burgdorferi* to evade humoral immunity.. Am J Pathol.

[14] Brown EL, Wooten RM, Johnson BJ, Iozzo RV, Smith A (2001). Resistance to Lyme disease in decorin-deficient mice.. J Clin Invest.

[15] Shi Y, Xu Q, Seemanapalli SV, McShan K, Liang FT (2006). The *dbpBA* locus of *Borrelia burgdorferi* is not essential for infection of mice.. Infect Immun.

[16] Blevins JS, Hagman KE, Norgard MV (2008). Assessment of decorin-binding protein A to the infectivity of *Borrelia burgdorferi* in the murine models of needle and tick infection.. BMC Microbiol.

[17] Xu Q, Seemanaplli SV, McShan K, Liang FT (2007). Increasing the interaction of *Borrelia burgdorferi* with decorin significantly reduces the 50 percent infectious dose and severely impairs dissemination.. Infect Immun.

[18] Xu Q, McShan K, Liang FT (2008). Essential protective role attributed to the surface lipoproteins of *Borrelia burgdorferi* against innate defences.. Mol Microbiol.

[19] Liang FT, Yan J, Mbow ML, Sviat SL, Gilmore RD (2004). *Borrelia burgdorferi* changes its surface antigenic expression in response to host immune responses.. Infect Immun.

[20] Purser JE, Lawrenz MB, Caimano MJ, Howell JK, Radolf JD (2003). A plasmid-encoded nicotinamidase (PncA) is essential for infectivity of *Borrelia burgdorferi* in a mammalian host.. Mol Microbiol.

[21] Schulze RJ, Zuckert WR (2006). *Borrelia burgdorferi* lipoproteins are secreted to the outer surface by default.. Mol Microbiol.

[22] Xu Q, McShan K, Liang FT (2008). Modification of *Borrelia burgdorferi* to overproduce OspA or VlsE alters its infectious behavior.. Microbiology in press.

[23] Coburn J, Fischer JR, Leong JM (2005). Solving a sticky problem: new genetic approaches to host cell adhesion by the Lyme disease spirochete.. Mol Microbiol.

[24] Cabello FC, Godfrey HP, Newman SA (2007). Hidden in plain sight: *Borrelia burgdorferi* and the extracellular matrix.. Trends Microbiol.

[25] Barthold SW, Hodzic E, Tunev S, Feng S (2006). Antibody-mediated disease remission in the mouse model of Lyme borreliosis.. Infect Immun.

[26] Johnson RC, Marek N, Kodner C (1984). Infection of Syrian hamsters with Lyme disease spirochetes.. J Clin Microbiol.

[27] Cox DL, Akins DR, Bourell KW, Lahdenne P, Norgard MV (1996). Limited surface exposure of *Borrelia burgdorferi* outer surface lipoproteins.. Proc Natl Acad Sci U S A.

[28] Liang FT, Caimano MJ, Radolf JD, Fikrig E (2004). *Borrelia burgdorferi* outer surface protein (*osp*) *B* expression independent of *ospA*.. Microb Pathog.

[29] Labandeira-Rey M, Seshu J, Skare JT (2003). The absence of linear plasmid 25 or 28-1 of *Borrelia burgdorferi* dramatically alters the kinetics of experimental infection via distinct mechanisms.. Infect Immun.

[30] Xu Q, Seemanapalli SV, Lomax L, McShan K, Li X (2005). Association of linear plasmid 28-1 with an arthritic phenotype of *Borrelia burgdorferi*.. Infect Immun.

[31] Reed LJ, Muench H (1938). A simple method of estimating fifty percent endpoint.. Am J Hygiene.

[32] Xu Q, Seemanapalli SV, McShan K, Liang FT (2006). Constitutive expression of outer surface protein C diminishes the ability of *Borrelia burgdorferi* to evade specific humoral immunity.. Infect Immun.

[33] Xu Q, McShan K, Liang FT (2007). Identification of an *ospC* operator critical for immune evasion of *Borrelia burgdorferi*.. Mol Microbiol.

