# Supplementary figures and images for: Common and Unique Contributions of Decorin-Binding Proteins A and B to the Overall Virulence of Borrelia burgdorferi
Source: PLoS One. 2008 Oct 3;3(10):e3340. doi: 10.1371/journal.pone.0003340 (PMC2556102; doi:10.1371/journal.pone.0003340)

A

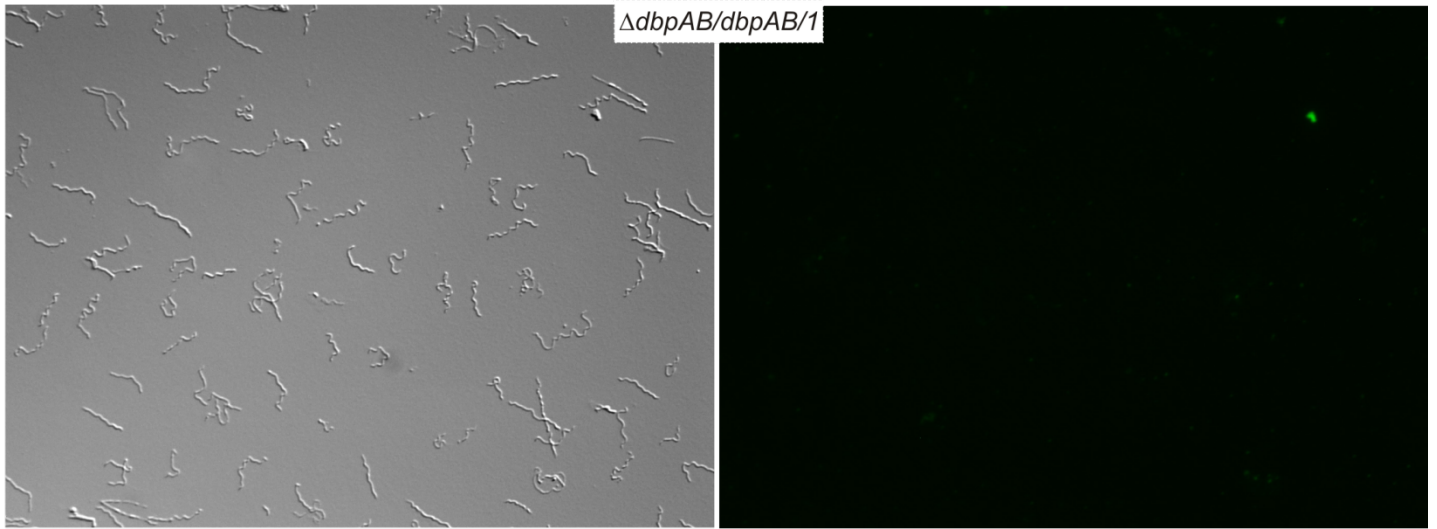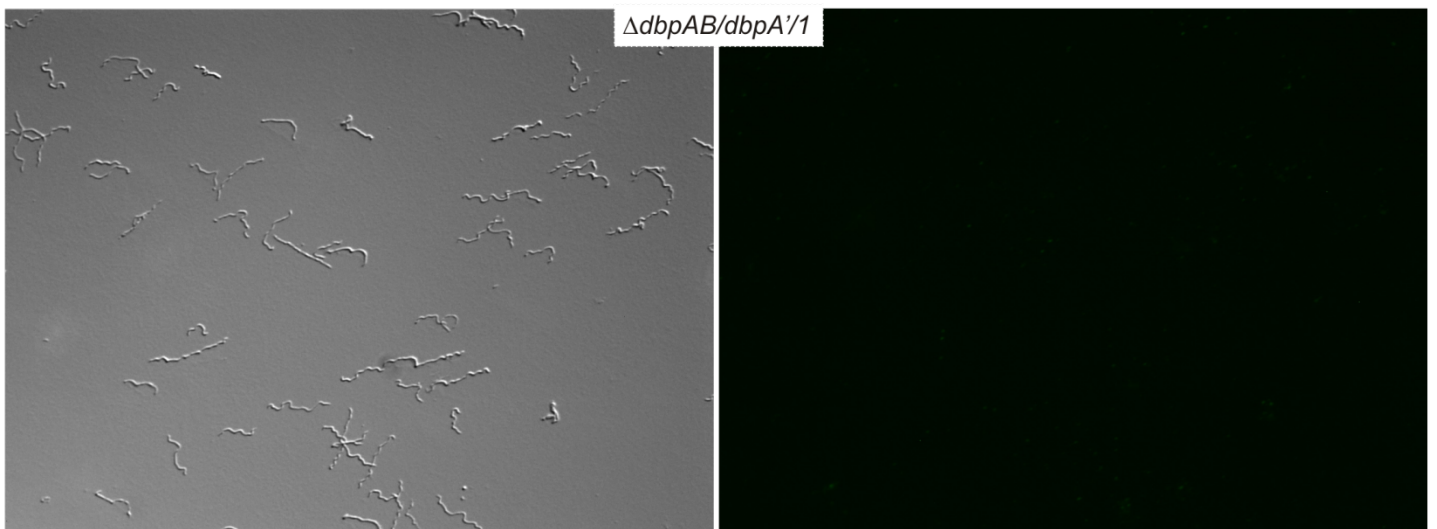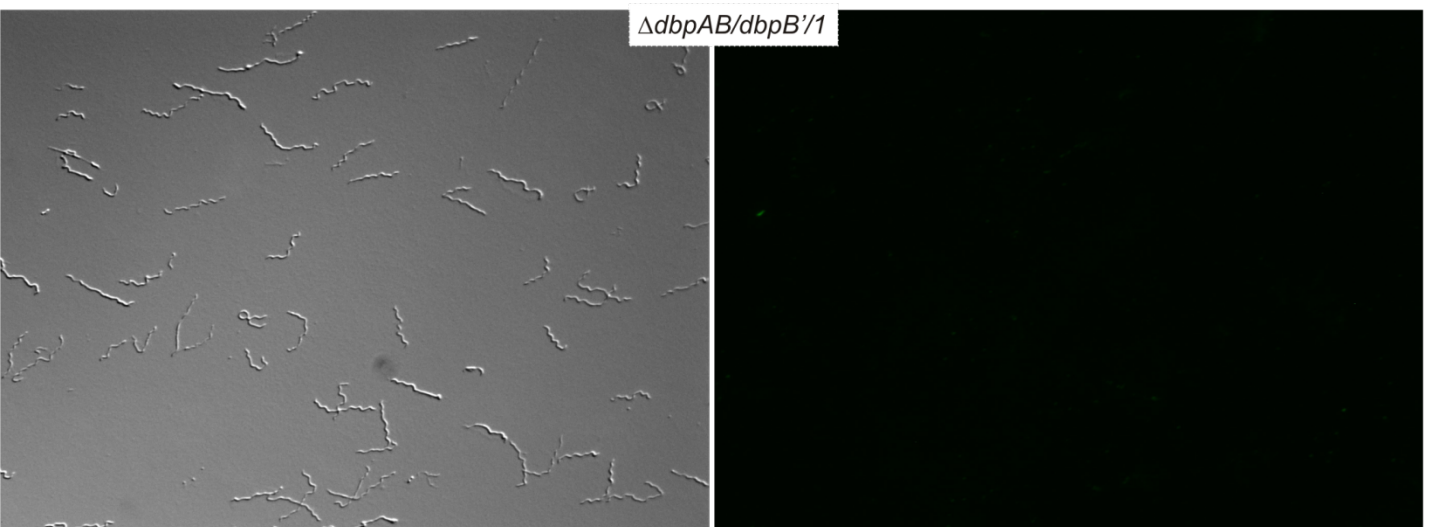

B

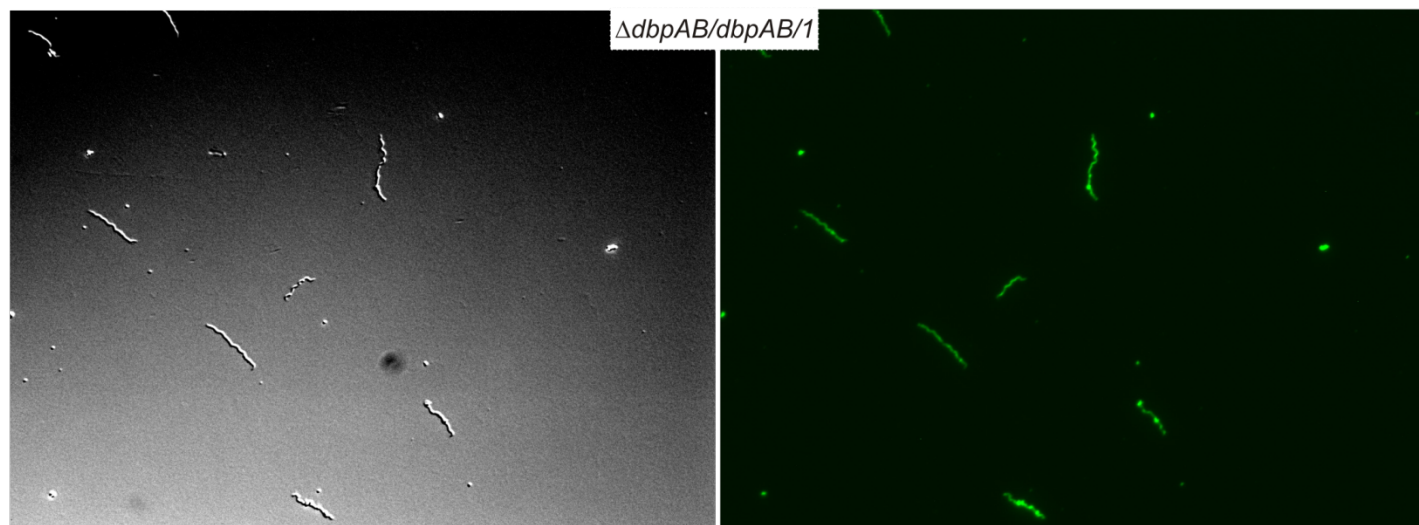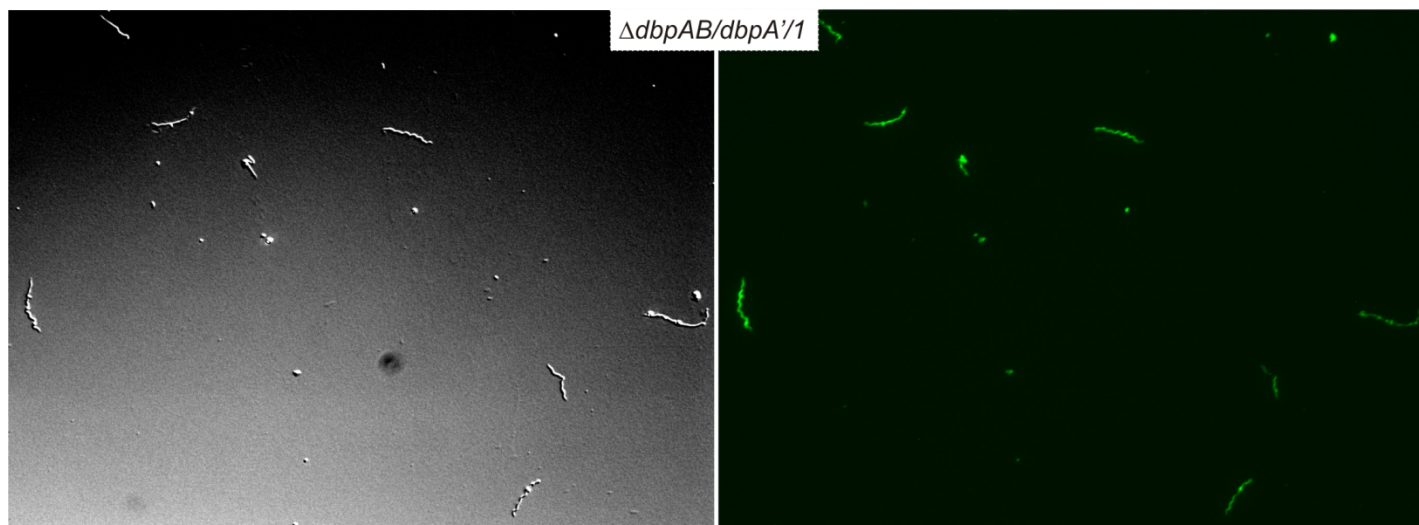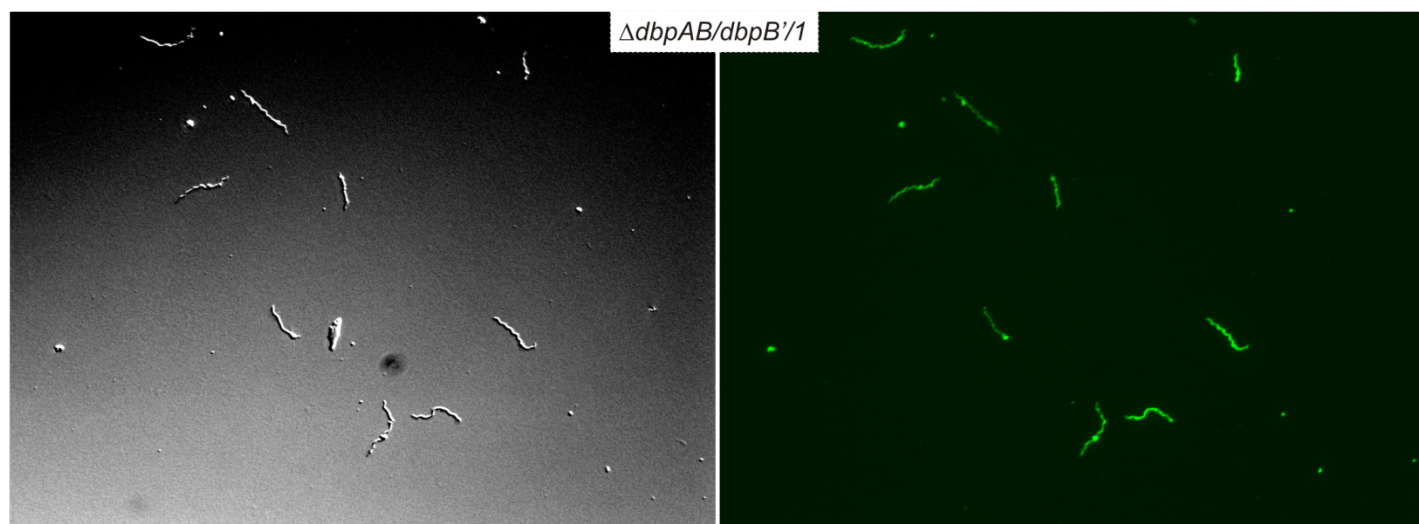

C

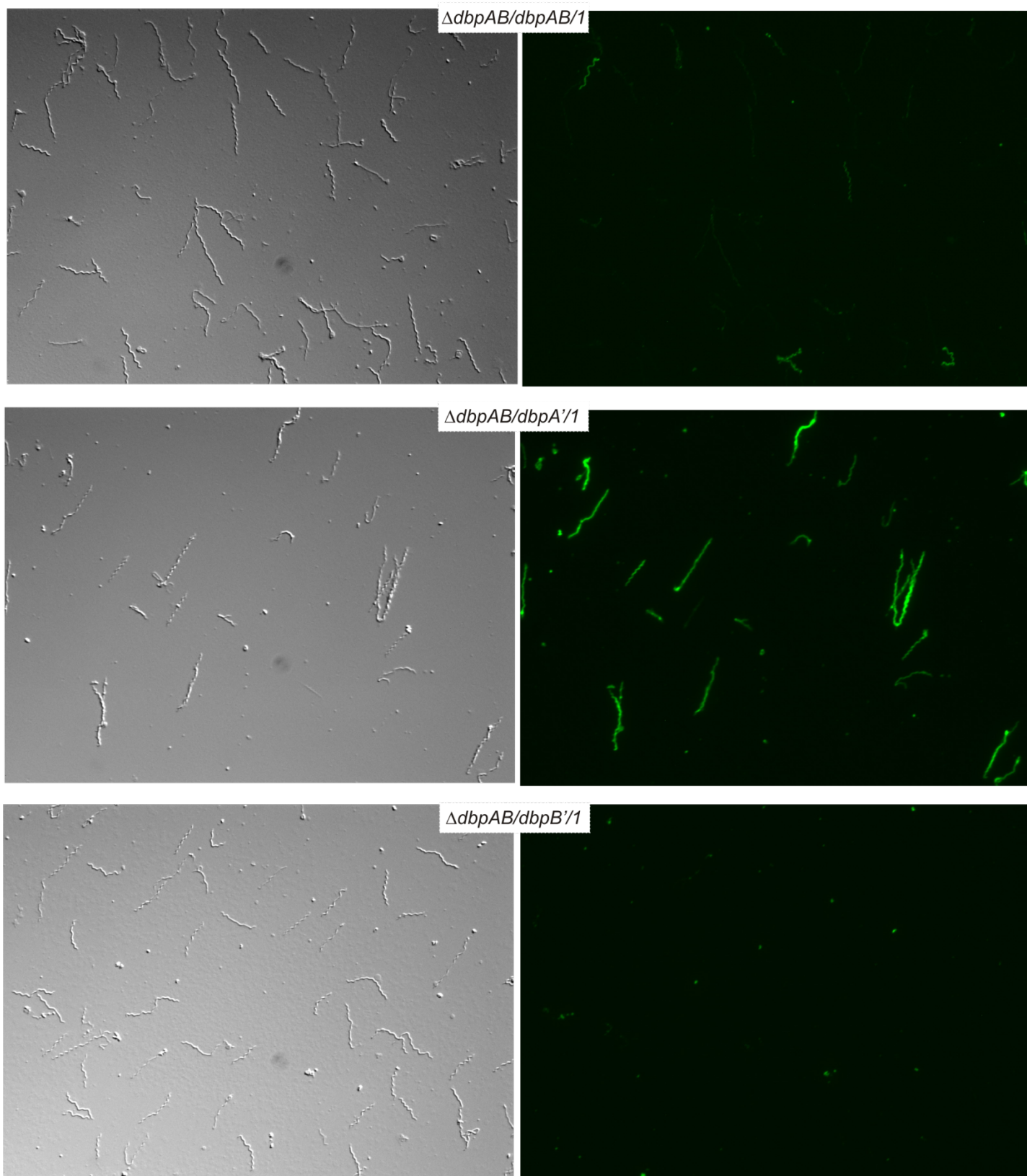

D

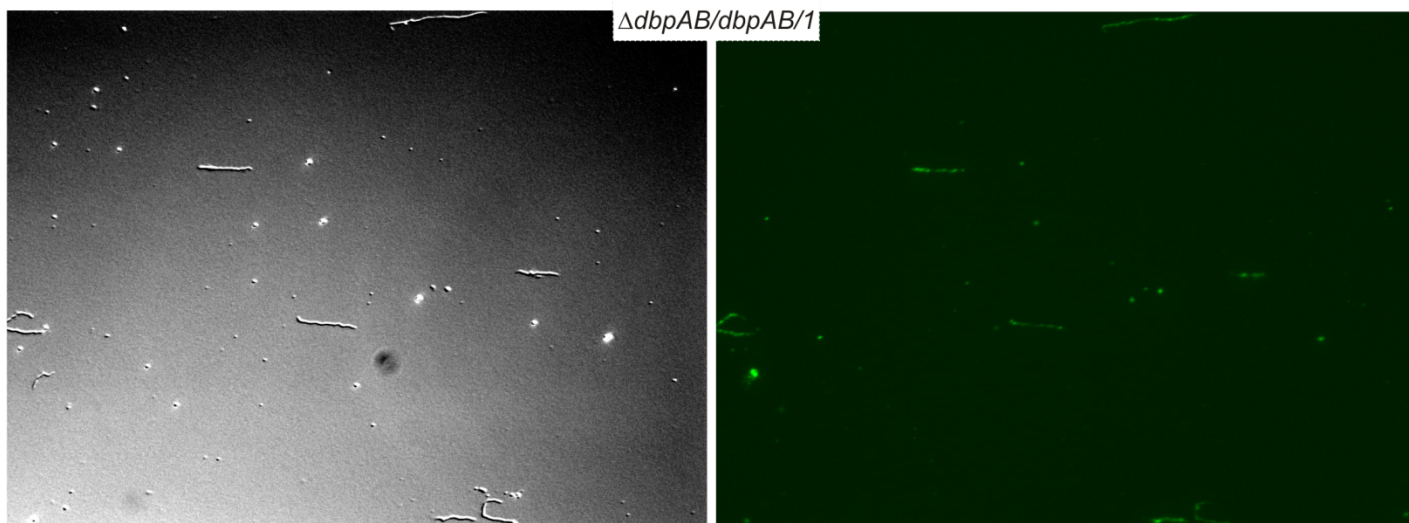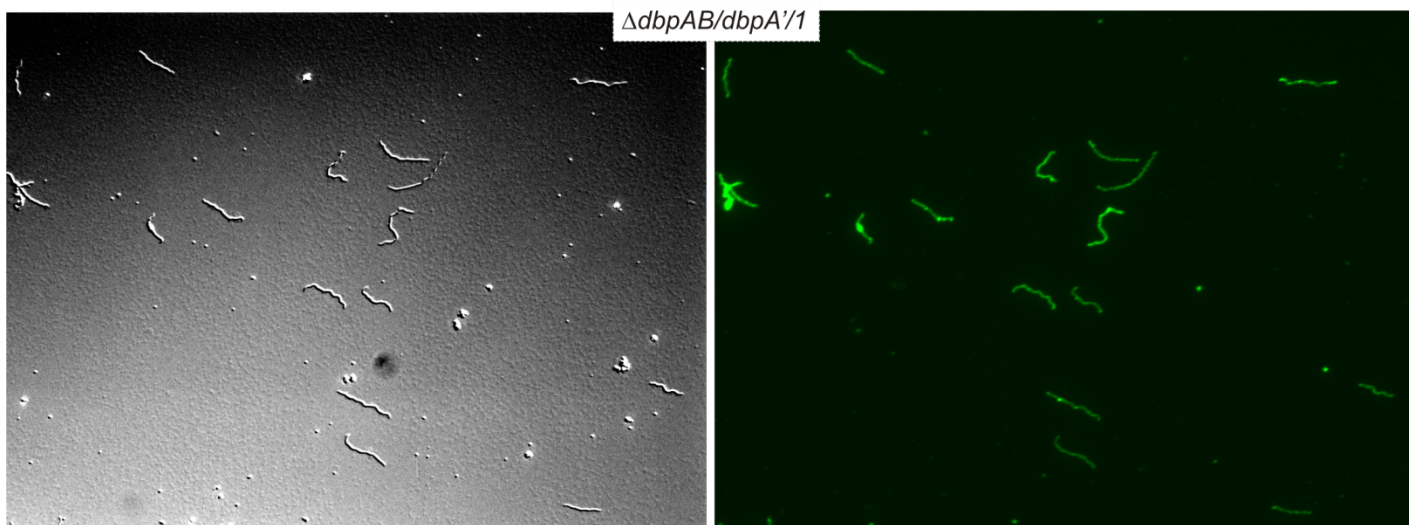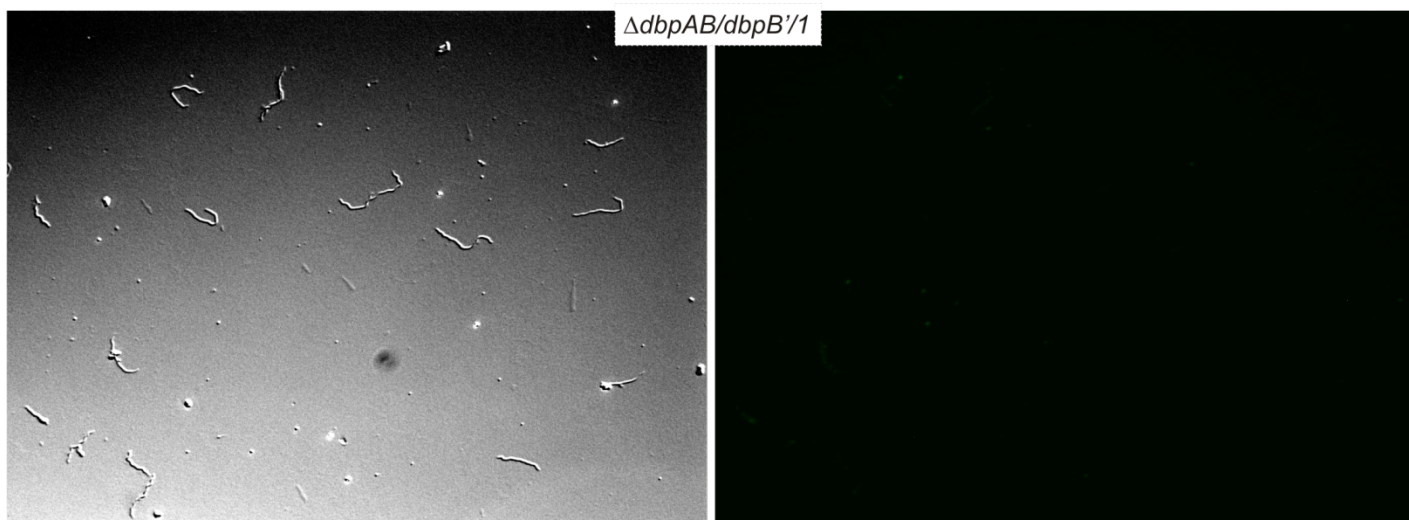

E

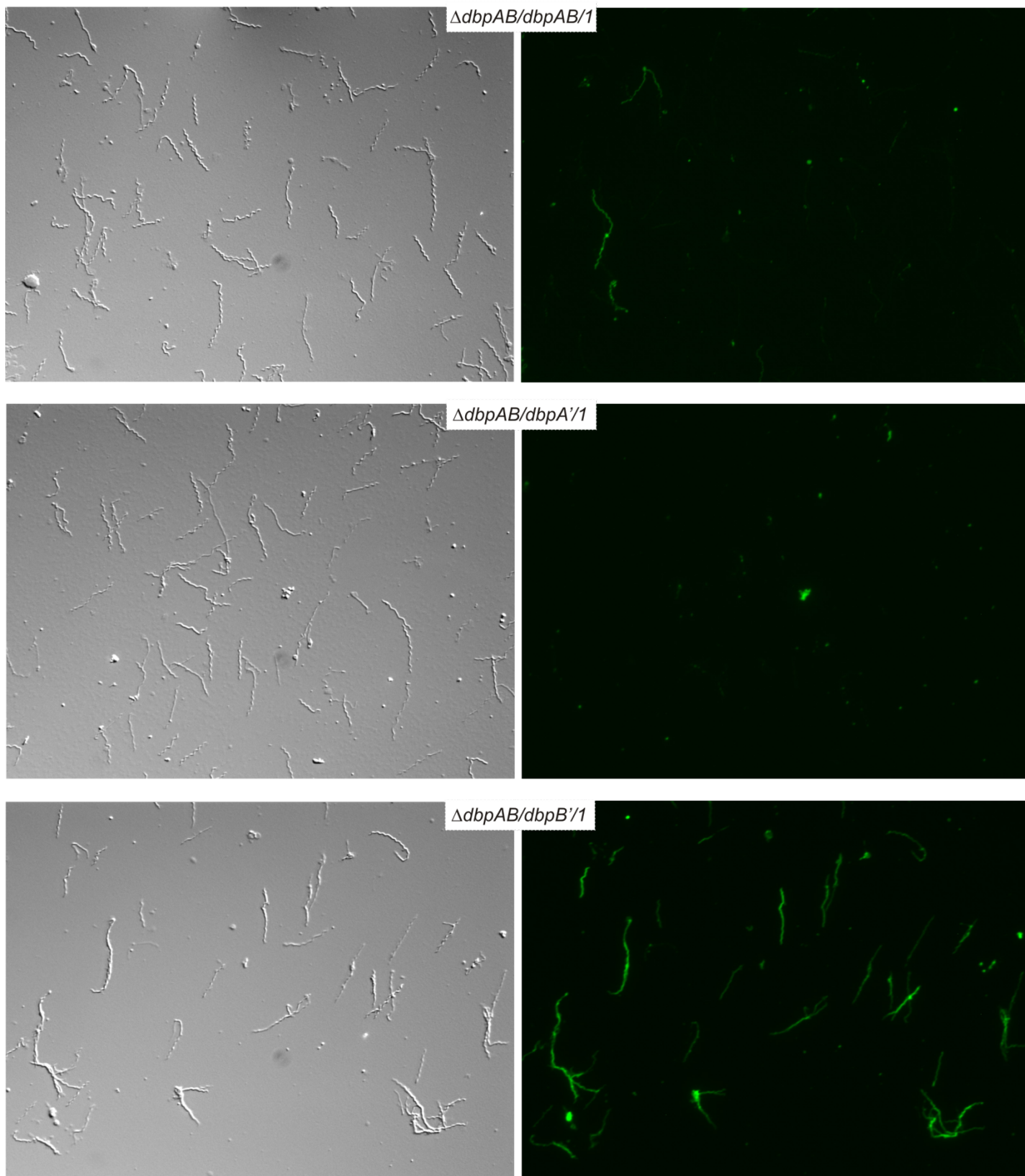

F

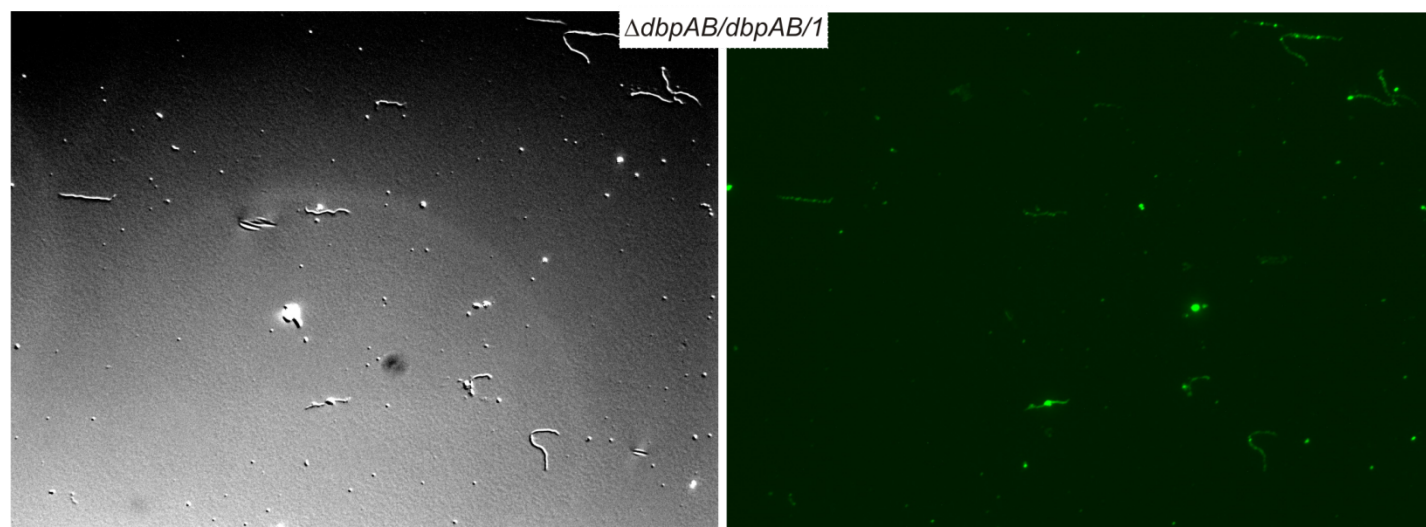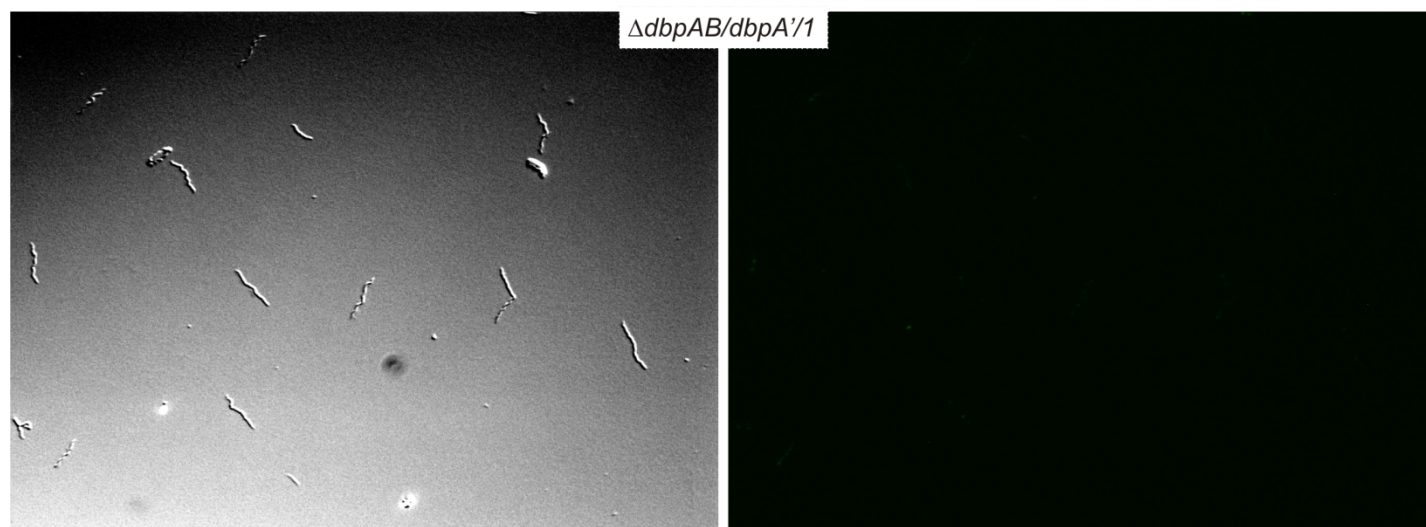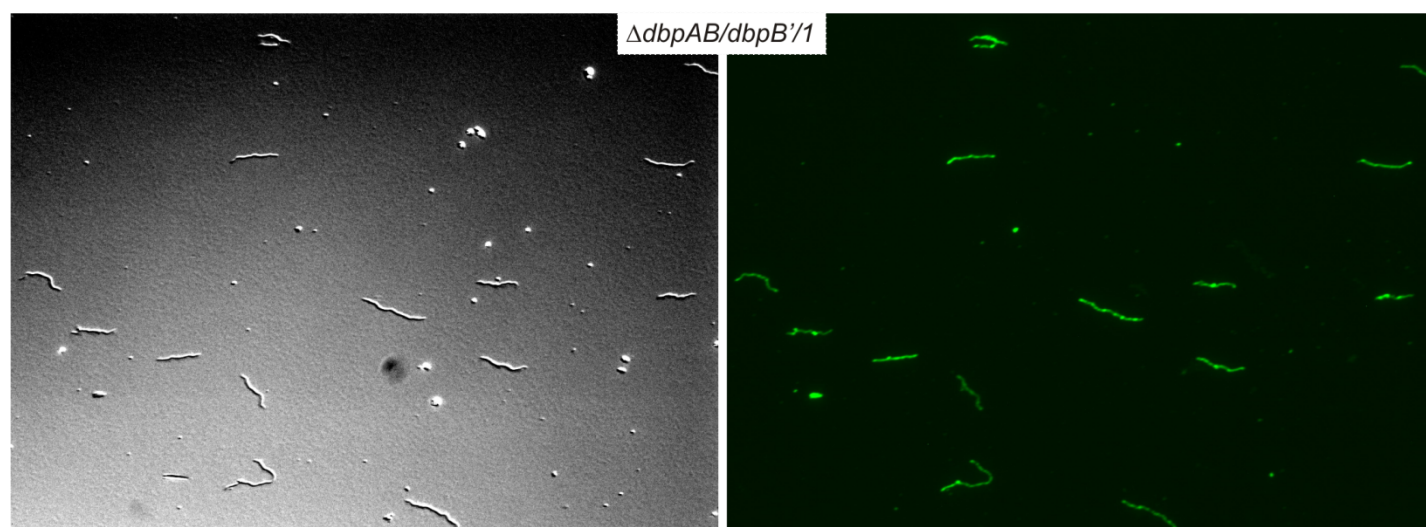

Supplement: Figure S1 — (13.57 MB PDF) [file pone.0003340.s001.pdf]
